# Variations in odontological care routines for patients undergoing treatment for head and neck cancer in county councils/regions of Sweden

**DOI:** 10.1002/cre2.242

**Published:** 2019-09-19

**Authors:** Niklas Bohm, Charlott Karlsson, Jessica Skoogh Andersson, Annica Almståhl

**Affiliations:** ^1^ Department of Oral Microbiology and Immunology, Institute of Odontology, Sahlgrenska Academy University of Gothenburg Gothenburg Sweden; ^2^ Clinic of Oral and Maxillofacial Surgery, Department of Orofacial Medicine, Institute of Odontology Jönköping University Jönköping Sweden; ^3^ Department of Periodontology, Institute of Odontology, Sahlgrenska Academy University of Gothenburg Gothenburg Sweden

**Keywords:** dental hygienist, dentist, oral cancer, oral care, questionnaire

## Abstract

**Objective:**

To investigate current odontological care routines for patients treated for head and neck cancers in the county councils/regions (C/Rs) of Sweden.

**Methods:**

An invitation to fill in a web‐based questionnaire was sent to dentists/dental hygienists working in dental clinics in the 12 C/Rs, treating and responsible for the odontological care of patients undergoing treatment for cancer of the head and neck. The questionnaire started with two mandatory and one non‐mandatory questions, followed by questions regarding routines before (*n* = 28), during (*n* = 23), and after (*n* = 9) treatment, plus two additional questions, totalling 65 questions.

**Results:**

Four dental hygienists and six dentists in 10 of the 12 C/Rs answered the questionnaire. Three C/Rs stated that they measure both the unstimulated and stimulated salivary secretion rate, and another C/R stated that they measure the stimulated secretion rate only. Similar recommendations were given regarding oral hygiene, salivary stimulants and substitutes, and extra fluoride. However, great variations were seen regarding recommendations for preventing and relieving oral mucositis. There were also discrepancies regarding information about the importance of avoiding smoking and alcohol. In seven C/Rs, patients visited the dental hygienist once a week during cancer treatment.

**Conclusion:**

The results suggests that there are great variations in odontological care given to patients undergoing treatment for cancer of the head and neck region in different county councils/regions in Sweden. There is a need to develop and implement evidence‐based guidelines to decrease the risk of oral complications and increase both the quality of life and the quality of care.

## 1 INTRODUCTION

Approximately 1,200 new cases of cancer of the head and neck region are diagnosed every year in Sweden (The Swedish National Board of Health and Welfare), with approximately 264,000 cases worldwide. (Jemal et al., 2011) Cancer of the head and neck region is twice as common among males compared with females. (Gupta, Johnson, & Kumar, 2016) Radiotherapy is a common treatment and is often combined with chemotherapy or surgery.

The treatment of cancer can lead to many side effects, which can be either acute/early or late. Acute/early side effects occur during or immediately after the treatment and are, for example, pain in the head and neck region, (Epstein et al., 2010) trismus, (Scott, D'Souza, Perinparajah, Lowe, & Rogers, 2011) oral mucositis, (Sroussi, Epstein, & Bensadoun, 2017) and reduced salivary flow/xerostomia, (Burlage, Coppes, Meertens, Stokman, & Vissink, 2001) which in turn may cause difficulty to speak and swallow and may also affect the sense of taste and smell.

Approximately 80–100% of patients treated for cancer of the head and neck area are affected by oral mucositis. (Sroussi, Jessri, & Epstein, 2018; Trotti et al., 2003) Oral mucositis, corresponding to Grade 3 (severe) or Grade 4 (life‐threatening) on the World Health Organization scale, arises in a high proportion of patients treated with high dose radiotherapy, and especially when radiotherapy is combined with chemotherapy(Trotti et al., 2003).

Late side effects of cancer treatment are often irreversible and may occur several months to years after completed radiotherapy, for example, trismus, dysphagia, osteoradionecrosis, low salivary secretion rate, permanent xerostomia, and caries. (Almståhl, Finizia, Carlén, Fagerberg‐Mohlin, & Alstad, 2018; Almståhl, Skoogh Andersson, Alstad, Fagerberg‐Mohlin, & Finizia, 2019; Epstein et al., 2012; Moon et al., 2017; Pauli, Johnson, Finizia, & Andréll, 2013)

It is of vital importance to prevent both acute and late complications due to cancer treatments. The Swedish National Care Program for Head and neck cancer(Regional Cancer Centres, Sweden, 2015) is sparse regarding odontological care routines before, during, and after cancer treatment. Several review articles have been published suggesting means and methods before, during, and after cancer treatment to prevent and/or relieve oral complications especially oral mucositis. (Buglione et al., 2016a; Buglione et al., 2016b; De Sanctis et al., 2016; Jensen et al., 2013; Lalla et al., 2014; McGuire et al., 2013; Nicolatou‐Galitis et al., 2013; Sroussi et al., 2017) However, as far as we know, no evidence‐based standard protocol regarding the care of these patients exists, and routines for collaborations between different healthcare professions involved in the care of this patient category is sparse. (Lanzós, Herrera, Lanzós, & Sanz, 2015; Moslemi et al., 2016; Sroussi et al., 2018) This may lead to large variations in the amount and/or quality of the odontological care given because it is up to the individual dental clinic to plan for the care of each patient, which may lead to inequalities in the care provided.

The aim of the present study was to investigate the current odontological care routines of dental clinics in county councils/regions in Sweden responsible for the care of this patient category being treated for head and neck cancers.

## 2 STUDY POPULATION AND METHODOLOGY

A web‐based questionnaire was developed aiming at investigating current odontological care routines for patients undergoing treatment for head and neck cancers before, during, and after treatment in the county councils/regions (C/R) of Sweden treating this patient category. The questions in the questionnaire were inspired by the study by Lanzós et al. (21), and by six documents/guidelines (in Swedish) regarding odontological care for patients with cancer of the head and neck. Two of the documents/guidelines were found on the internet(Oncology Centre Stockholm‐Gotland, 2007; Swedish Association of Orofacial Medicine (SOM), 2017) and four were obtained in paper form by personal communication. (Anonymous, 2015; Augustsson et al., 2006; Mellgren, 2017; Nilsson & Broberg, 2017) The web‐based questionnaire was developed on http://webbenkater.com.

Before the questionnaire was sent to the intended respondents, the questionnaire was reviewed by author C. K. and one other dental hygienist, both with experience of odontological care of patients with cancer in the head and neck region. The review led to the reformulation of 27 of the questions. Nine questions were excluded as well as some answer alternatives, because the reviewers thought it went beyond the responsibilities of dentistry. The final version of the questionnaire consisted of 65 questions (Supporting information).

The questionnaire started with two mandatory questions regarding which C/R the respondent worked in and his/her occupation, followed by a non‐mandatory question regarding how many patients were treated for cancer of the head and neck region each year in their respective C/R. The next part in the questionnaire concerned the odontological care routines before starting cancer treatment and consisted of 28 questions. Part 3 was about odontological care during cancer treatment and consisted of 23 questions, and Part 4 concerned odontological care after completed cancer treatment (nine questions). The questionnaire ended with two statements: “We have documented procedures for the entire odontological care” and “We have written patient information that we distribute.”

Thirty‐four questions were yes/no‐questions. For 18 questions, the respondent could choose to mark one or more of between two and 20 predetermined answers. These questions were, for example, professions involved in patient care, which oral complications the patients were informed of, advice to prevent/treat oral mucositis and relieving dry mouth. For 22 of the questions, there was an opportunity to comment on the answers given. Nine of the questions contained subqueries with the possibility to provide various answers. It was also possible to comment on all questions in free text.

An email was sent to dentists/dental hygienists involved in the odontological care of patients undergoing treatment for cancer of the head and neck region in the 12 different C/Rs (Table 1) in January 2018. The aim of the questionnaire study was presented and information that the estimated time to fill in the questionnaire was 35–40 min. The respondents were also informed that results were going to be presented on a C/R level and that no dental hygienist/dentist would be identifiable. A link to the web‐based questionnaire on http://webbenkater.com was also given. Two reminders were sent via email to respondents who had not answered the survey.

**Table 1:** The 12 county councils/regions from the North to the South, which treat patients with cancer of the head and neck region

County council/region	Answered the questionnaire
Västernorrland County Council	Dentist
County Council of Värmland	Dental hygienist
Region Uppsala	Dentist
Region Västmanland	Dentist
Stockholm County Council	Dentist
Region Örebro	No reply
Region Östergötland	Dentist
Region Västra Götaland	Dentist
Region Jönköping County	Dental hygienist
County Council of Kalmar	Dental hygienist
Region Kronoberg	No reply
Region Skåne	Dental hygienist

### 2.1 Statistical analysis

The C/Rs were randomly assigned a code from 1 to 10. Questions Number 8 and 9 about recommendations to relieve oral mucositis and oral mucositis pain, respectively, were merged.

## 3 RESULTS

Dentists (*n* = 6) and dental hygienists (*n* = 5) in 10 of the 12 C/Rs providing odontological care for patients undergoing radiotherapy treatment for cancers of the head and neck region completed the questionnaire (Table 1). Questions about before starting radiotherapy where the respondent could answer Yes or No and give a comment are presented in Table 2.

How many patients/year undergo treatment for cancer of the head and neck region in your C/R?

Eight of the 10 C/Rs answered this question and the number varied between 20 and 160.

**Table 2:** Distribution of answers to Yes/No questions regarding routines before treatment (Question Numbers 4, 6, 8–10, 14–17, 19, 21–23, 26–28)

Before treatment	Yes	No	No answer/other answer
*B4.* Is the patient asked about self‐perceived oral health during the examination?	70%	30%	0%
*B6.* Is an odontological treatment plan established so that the patient and all parties involved agree before the patient begins cancer therapy?	100%	0%	0%
*B8.* Is the patient's prosthetic construction, its design and function evaluated?	100%	0%	0%
*B9.* Is previous trauma assessed/considered?	90%	10%	0%
*B10a.* Are photographs taken prior to start of cancer therapy?	50%	10%	40%
*B10b.* Are dental impressions (alginate or similar) made prior to cancer therapy?	50%	40%	10%
*B14.* Do you have specific routines regarding treatment of carious lesions prior to cancer treatment?	0%	30%	70%
*B15.* Is the ability to open the mouth determined?	90%	10%	0%
*B16.* Do you have routines regarding reduced ability to open the mouth?	100%	0%	0%
*B17a.* Is the unstimulated salivary secretion rate measured?	30%	70%	0%
*B17b.* Is the stimulated salivary secretion rate measured?	40%	60%	0%
*B18.* Are impressions for customised trays taken?	40%	50%	10%
*B19.* Does the patient receive an individually adapted recommendation regarding extra fluoride?	90%	10%	0%
saliva stimulants?	60%	0%	40%
saliva substitutes?	70%	0%	30%
*B21.* Does the patient receive information and instruction regarding oral self‐care?	100%	0%	0%
*B22.* Is the patient given a motivational dialogue about optimal oral hygiene?	100%	0%	0%
*B23.* Does the patient receive instructions regarding toothbrushing technique?	90%	10%	0%
*B26.* Does the patient receive an individually adapted recommendation regarding interproximal oral self‐care aids?	80%	20%	0%
*B27.* Is the patient informed about the importance of abstaining from smoking and alcohol during cancer treatment?	50%	30%	20%
*B28.* Do you have special routines for patients with reduced ability to maintain adequate oral hygiene or patients with comorbidity?	30%	0%	70%

### 3.1 Before starting radiotherapy

B1. How is the contact with the dental care administrated?

In all C/Rs, a referral is sent to the dental clinic from the otorhinolaryngology clinic when a patient is planned to start cancer treatment. Nine C/Rs clarified that a referral is also sent to the dental clinic from the responsible oncologist, and four C/Rs that the dental clinic participates in a multidisciplinary conference.

B2. Which professions are involved in the care?

In all C/Rs, a dental hygienist, a dentist, a dental assistant, a physician, and a contact nurse are involved. In nine C/Rs a dietitian is also involved; in eight C/Rs a maxillofacial surgeon, a nurse, and a speech‐language pathologist are involved; and in seven C/Rs, social workers are also involved.

B3. What is recorded in the medical history when the patient visits the dental clinic?

All 10 C/Rs answered that they record oral discomfort and the name of the regular dental clinic. Nine C/Rs also record diseases, medicines, and allergies; eight record information about tobacco use; seven record dental anxiety; and three C/Rs ask about and record alcohol habits. One C/R also asked about the patient's social network. One C/R commented that they obtain information about diseases, medicines, allergies, tobacco use, and alcohol habits from the medical journal.

Seven C/Rs stated that they ask the patient about self‐perceived oral health (Question B4, Table 2).

B5. Is information about the risk of acute/temporary oral complications and actions to prevent or relieve them given in written form and/or orally?

Eight of the C/Rs stated that both written and oral information is given and two C/Rs answered orally. All C/Rs inform the patient about oral mucositis and xerostomia (Question B5b). Nine C/Rs also give information about pain and risk of fungal infections, eight C/Rs also give information about swallowing difficulties and impact on sense of taste and smell, seven C/Rs give information about reduced appetite, six C/Rs give information about speech difficulties, weight loss, and fatigue/exhaustion. Six of the C/Rs provided additional answers: “We strive to provide the patient with information related to the oral health and the physicians provide information related to the general health,” “We provide the patient continuously with bits of information and not all at once,” “The overall first information is given by the contact nurse and they provide the patient with more information as the problems eventually occur,” “The information first and foremost is provided at the oncology clinic,” “We inform the patient about reduced ability to open the mouth and sticky saliva,” “The patient receives information from a lot of different professions, mainly physicians.”

In all C/Rs, an odontological treatment plan is established, which the patient and others involved agree with before the patient begins cancer therapy (Question B6, Table 2). One C/R commented their answer: “We write our odontological treatment plan in the same journal system as the healthcare system so that its personnel can take part of it.”

B7. What kinds of examinations are performed before cancer treatment?

X‐ray: Nine C/Rs answered panoramic X‐ray, eight C/Rs added full status, seven C/Rs answered that they also take bitewing X‐rays. Three C/Rs answered that they sometimes use cone beam computed tomography (CBCT). One C/R request the opinion from the Department of Oral Radiology. One C/R supplements the examination with Orthopantomograph (OPG) and apical X‐rays when needed. One C/R considered computed tomography (CT‐scan) when a full status X‐ray was not possible.
Periodontal status: Nine C/Rs measure pocket probing depth, furcation involvement, mobility, and periradicular status. One C/R only measures pocket probing depth.
Cariological status: All C/Rs answered that they use a probe to examine caries status and that they take bitewing X‐rays. One C/R added that they also shine light through the teeth.

B8. Are the patient's prosthetic constructions, its design, and function evaluated? (Table 2)

One C/R commented their answer: “We evaluate the prosthetics if needed in collaboration with the patient's regular dental clinic, or if needed we contact a specialist in prosthodontics.”

B9. Is previous trauma assessed/considered? (Table 2)

The majority of the C/Rs take previous trauma into consideration. Three C/Rs commented their answer: “We usually ask for the X‐rays from the patient's regular dentist,” “We assess the trauma if it is important for the planned treatment,” “We assess trauma if it is brought up by the patient.”

B10. Are photographs taken prior to start of cancer therapy? (Table 2)

Five C/Rs take photographs. Four C/Rs gave comments: “If necessary” or “sometimes” (three C/Rs) and “If there is an indication” (one C/R).

B10b. Are dental impressions (alginate or similar) made prior to cancer therapy?

Eight C/Rs commented their answer: “When needed, for example prior to producing a tongue‐depressor,” “If a dental splint or a mouth opening device is to be produced,” “We make customised trays for fluoride gel for all patients,” “Rarely, only when needed,” “Dental impressions are only taken for prophylactic measures on patients with extensive prosthetic constructions (crowns, bridges), or for patients with active caries,” “Prior to eventual surgery/resection, and when in need of for example palatal plate, splint etc.,” “We are about to start with it” and “It depends on the diagnosis and planned medical treatment.”

B11. On which indications are teeth extracted? (Table 3)

**Table 3:** Question B11: On which indications are decisions on tooth extraction based? All C/Rs extracted a tooth with deep periodontal pockets, vertical bone defects, furcation involvement, periradicular changes, and apical changes. Indications given by only one C/R were dental status, dental habits, nausea, partially erupted teeth, pericoronitis, tooth mobility, acute symptoms, fractures, and curative/palliative radiation therapy

Indication	C/R1	C/R2	C/R3	C/R4	C/R5	C/R6	C/R7	C/R8	C/R9	C/R10
Caries					x	x		x	x	
Root residue		x						x		
Radiation dose					x		x	x		
Spreading of radiation field					x		x	x	x	
Risk of osteoradio‐necrosis					x		x			
Infections	x	x	x							x
Teeth in radiation field with uncertain prognosis									x	x
Damaged teeth		x		x						
Doubtful endodontic prognosis								x	x	

All C/Rs stated that periodontal problems such as deep pockets, vertical bone defects, furcation involvements, periradicular changes, and apical changes were indications for tooth extraction. Four C/Rs also stated caries, infections, and the radiation field as indications for extraction (Table 3).

B12. How long before the start of the cancer treatment are the extractions performed?

The most common answers were 2 weeks or 2–3 weeks (six C/Rs) and 7–10 days or 10 days (two C/Rs).

B13. On which indications are caries treatment performed?

Nine of the 10 C/Rs answered the question and six of those C/Rs answered that manifest caries and carious lesions close to the pulp were indications for caries treatment. One C/R answered that they always ask the patient's regular dentist to perform dental restorations on all manifest caries lesions before the radiotherapy starts. One C/R answered: “Absence from infection in the radiation area” and one C/R wrote “It is important to create as healthy oral conditions as possible.”

B14. Do you have specific routines regarding treatment of carious lesions prior to cancer therapy? (Table 2)

Three C/Rs answered “No” and one C/R answered that they did not understand the question. Seven C/Rs each gave different comments: “Sometimes temporary fillings are performed, alternatively caries treatment early in the radiation treatment schedule. It is sometimes difficult to judge if the carious lesion is not close to the pulp, which means that the patient him/herself has to pay for the fillings, which might be problematic if they have poor economy,” “We strive to treat all deeper cavities if there is time; otherwise, we do temporary fillings,” “We prioritize structures in the field of radiation,” “Temporary fillings are compensated by the ordering unit,” “We follow the instructions from the ordering unit, permanent fillings are accepted,” and “Depending on the amount of time at hand, the patient is offered caries treatment prior to start of cancer treatment.”

B15. Is the ability to open the mouth determined? (Table 2)

All but one C/R agreed that the patient's ability to open the mouth should be measured. Three C/Rs commented their answers: “Measurement of mouth opening is performed at every visit to the dental hygienist,” “We only measure mouth opening on patients with a marked impaired ability to open the mouth,” and “We document the ability to open the mouth both prior to, and post radiation treatment.”

B16. Do you have routines regarding reduced ability to open the mouth? (Table 2)

All C/Rs answered that they have such routines. Two comments were given: “We supply the patient with a jaw‐exercise program and inform about training aid products, for example Jaw Trainer” and “We have postoperative radiation therapy routines.”

B17ab. Is the patient's unstimulated and/or salivary secretion rate measured? (Table 2)

Three of the C/Rs measure both the unstimulated and the stimulated salivary secretion rate.

B18. Are impressions for customised trays taken, and on what indications? (Table 2)

Among the four C/Rs who make impressions, the indications were the following: caries prophylaxis (*n* = 2), large prosthetic constructions (*n* = 1), and abrasions on the tongue if smoothening of cusps does not help (*n* = 1).

B19. Does the patient receive an individually adapted recommendation regarding extra fluoride (a), saliva stimulants (b), and saliva substitutes (c)? (Table 2)

Nine C/Rs give individually adapted recommendations regarding extra fluoride, most frequently rinsing with 0.2% sodium fluoride twice a day (five C/Rs), 2–3 times per day (one C/R), and once daily (three C/Rs). Of the six C/Rs who answered the question about saliva stimulates, five recommended Xerodent (tablet containing malic acid, sodium fluoride, and xylitol) either “several times per day,” “six times per day,” or “when needed” and other tablets or chewing gum were recommended by one C/R each. One C/R recommend Salagen (pilocarpine) prescribed by a doctor. Of the seven C/Rs who answered the question about saliva substitutes, the most frequently recommended products were Proxident spray (sun‐flower oil; five C/Rs) and a saliva substitute prepared at the pharmacy (containing sodium chloride, sodium fluoride, xylitol; three C/Rs).

B20. What dental treatments are given?

Eight C/Rs answered the following: “Scaling,” “Polishing of filling joints,” and “Smoothening of sharp cusps and incisor edges.” One C/R answered “As needed before radiotherapy” and one “Oral hygiene optimization, interventions according to individual needs.”

All C/Rs stated that the patient receives information and instructions regarding oral self‐care (Question B21, Table 2), and all stated that a motivational dialogue about the importance of optimal oral hygiene is given (Question B22, Table 2).

All but one C/R stated that the patient receives instructions regarding toothbrush technique (Question B23, Table 2). Seven C/Rs recommend a soft or extra soft toothbrush (Question B24). Two C/Rs answered “Individual recommendation based on the patient's needs” and two “Electrical toothbrush.”

The C/Rs agreed that toothpaste without sodium lauryl sulphate should be recommended (Question B25). The importance of recommending a toothpaste with no/mild taste was stated by three C/Rs. Eight of the C/Rs stated that the patient receives an individualized recommendation regarding interproximal oral self‐care aids (Question B26, Table 2).

In five of the C/Rs, the patient is informed about the importance of abstaining smoking and alcohol (Question B27, Table 2). Several comments were given: One C/R answered that “The patient is informed by the oncologist and contact nurse,” one C/R that they “Adapt the information for when it is most suitable for the patient.” Three C/Rs answered that they inform about the importance of smoking cessation but not about the importance of abstaining alcohol. Two C/Rs clarified that it is up to the physicians to have the discussion about alcohol with the patient.

Regarding patients with decreased ability to maintain an adequate oral hygiene or patients with comorbidity, only three C/Rs stated that they have special routines (Question 28, Table 2).

### 3.2 During radiotherapy

D1. How often does the patient meet different professionals during the treatment?

Seven C/Rs stated that the patient visited the dental hygienist once a week and the other three C/Rs answered the following: “It varies,” “When needed,” and “On individual basis.” The patient visited the dentist “Once a week” in one C/R, “1–2 times” in two C/Rs, “When needed” in six C/Rs, and not at all in one C/R. There was also great variation regarding how often the patient visits health care personnel during treatment. Most frequently reported were visits to the doctor (six C/Rs, from three visits to once a week during RT), dietitian (five C/Rs, once to once a week during RT).

All C/Rs stated that they re‐inform the patient about the effect of radiation on oral health (Question D2). Eight C/Rs answered that the oral cavity is inspected by dental personal once a week, one C/R said that “the oral care round is responsible,” and the last C/R answered “individual” (Question D3). Other professions who inspect the oral cavity were: doctor (all C/Rs), nurse (six C/Rs), and speech therapist (four C/Rs; Question D4).

Nine of the C/Rs stated that signs of possible mucositis or other signs of abnormal oral health are noted at the inspections of the oral cavity (Question D5). The World Health Organization scale was most commonly used for grading mucositis (eight C/Rs; Question D6). One C/R used the Radiation Therapy Oncology Group scale, one the the Oral Mucositis Assessment Scale, and one C/R did not answer the question. One C/R added that they supplement the scale grading with photographs.

D7a. What means are recommended to prevent mucositis?

Seven C/Rs recommend good/optimal oral hygiene (in five of those C/Rs, the patient visited the dental hygienist once a week), two C/Rs added the importance of moistening and lubricating the oral mucosa, and two C/Rs recommend saline and one saliva substitute. One C/R answered that recommendations regarding prevention of mucositis is given by the pain management unit at the hospital. One C/R added that smoking and alcohol cessation is important. One C/R answered that it is not possible to prevent oral mucositis because it is a complication of the radiotherapy.

D7b. What/which preparations are recommended to prevent mucositis?

Nine of the 10 C/Rs answered this question. The most frequent recommendations were the following: rinsing with saline (six C/Rs), Lidocaine hydrochloride in oral cleaner (glycerol 85%, sodium chloride, polysorbate 40, peppermint oil, water; four C/Rs), oral cleaner (two C/Rs), and bromhexine hydrochloride (two C/Rs). Other recommendations were benzydamine oral rinse, tap water, and chlorhexidine (each recommended by one C/R). Four C/Rs added additional answers whereof two C/Rs answered “It is not possible to prevent oral mucositis since it is a side‐effect of radiation.” One C/R answered that they recommend the patient to rinse the mouth with mineral water after each meal and one that they recommend Caphosol (EUSA Pharma; oral rinse for moisturizing, lubricating, and cleansing of the oral cavity).

D8ab. What means are recommended to relieve mucositis problems and pain and advice regarding pain relief? (Table 4)

**Table 4:** Answers to Question D8. What means are recommended to relieve oral mucositis/pain due to oral mucositis? Means reported by only one C/R were: Nystimex, Cortisone, Lidocain‐morphine gel, Proxident sunflower oil spray, Mineral water, extra soft toothbrush, mild toothpaste, cream, homemade saliva substitute, rinsing the mouth after a meal, and assisted oral hygiene

Means recommended	C/R1	C/R2	C/R3	C/R4	C/R5	C/R6	C/R7	C/R8	C/R9	C/R10
Lidocainhydrochloride in oral cleaner	x	x	x	x	x	x	x	x		x
Analgetics prescribed by doctor	x	x		x			x	x		x
Sodiumchloride				x	x					x
Benzydamine	x	x								
Xylocain spray						x				x
Lidocain ointment	x		x	x	x					
Avoid warm and spicy food	x	x								
Good oral hygiene		x					x			

The most common recommendations were Lidocaine hydrochloride oral topical solution (nine C/Rs) followed by “doctor‐prescribed analgesics, for instance morphine” (six C/Rs), and four C/Rs stated Lidocaine ointment. Three C/Rs answered “rinse the mouth with saline.” A number of other recommendations were also added by single C/Rs (Table 4).

D9. What information, guidelines, or research do you base the pain relief treatment regarding oral mucositis on?

All regions gave different answers to this question. Examples of the documents specified (all in Swedish) were “drugs in the dental practice,” online recommendations by the Swedish Association for Orofacial Medicine/SOM, (Swedish Association of Orofacial Medicine (SOM), 2017) and regional/national guidelines. Three C/Rs said “proven experience.”

D10. Are dietary advice/recommendations given? (Table 5)

**Table 5:** Answers to Yes/No questions about routines during treatment (Nos‐ 10, 12‐14, 16‐23)

Questions: during treatment	Yes	No	No answer/other answer
*D10.* Are dietary advice/recommendations given?	10%	50%	40%
*D12.* At suspected mucosal infections, are samples for microbial analysis taken?	40%	40%	20%
*D13.* Is the fluoride prophylaxis followed up?	90%	10%	0%
*D14.* Is the salivary secretion rate measured during treatment?	50%	20%	30%
*D16.* Is the patient given written information about the recommendation of dry mouth products?	80%	0%	20%
*D17.* Is a reinstruction regarding toothbrushing technique given?	70%	30%	0%
*D18.* Is the patient given re‐information about recommended toothbrush?	60%	20%	20%
*D19.* Is the patient given re‐information about toothpaste?	90%	10%	0%
*D20.* Is the patient given re‐information about the recommended interproximal devices?	70%	20%	10%
*D21.* Is the patient given information, instruction and motivation to do mouth‐opening exercises?	90%	0%	10%
*D22.* Is the patient re‐informed about the importance of abstaining smoking and alcohol?	60%	40%	0%
*D23.* Information about the risk of late/chronic complications and means to relieve them?	100%	0%	0%

Only one C/R stated that they give advice or recommendations regarding diet. All C/Rs stated that the dietitian gives dietary advice and four C/Rs added the doctor (Question D11). Comments given were the following: “The dietitian handles this” (*n* = 4), “Individual, many patients cannot eat at all during parts of the cancer treatment,” and “Rinsing after intake of nutritional supplements. Patients getting nutritional support are informed about the importance of good oral hygiene. It is difficult with recommendations since it is important to get as much nutrients as possible.”

Four C/Rs stated that microbial samples are taken at suspected mucosal infections (Question D12, Table 5), four said “No,” one answered “Sometimes,” and one “Don't know.”

D13. Is the fluoride prophylaxis followed‐up? (Table 5)

All but one C/R followed up the fluoride prophylaxis and the last C/R stated that this was done by the ordinary dentist.

D14. Is the patient's salivary secretion rate measured during treatment? (Table 5)

Five of the C/Rs stated that they measure the salivary secretion rate during and two C/Rs were the same as those stating that they measured both the unstimulated and the stimulated salivary secretion rate also before treatment. Two C/Rs did not answer yes or no, but answered that “this is done 6–12 months post treatment” and one C/R answered that “the ordinary dentist measures the salivary secretion rate.”

D15. Which products are recommended at dry mouth?

All C/Rs recommended different moisturizing gels or sprays and saliva‐stimulating spray. Seven C/Rs recommended sugar‐free chewing gum. Eight C/Rs recommended different kinds of tablets developed for persons with dry mouth problems. All C/Rs recommended toothpastes with no sodium lauryl sulphate and/or developed especially for dry mouth patients (i.e., mild taste). Seven of the C/Rs stated that the patients receive written information about the recommended products (Table 5) and one C/R stated that this sometimes occurred (Question D16).

D17. Is a reinstruction regarding toothbrushing technique given? (Table 5).

Seven C/Rs stated that the patient's ability to brush the teeth was followed‐up and a reinstruction is given when needed.

Re‐information about recommended toothbrush and toothpaste (D18 and D19, Table 5)

Nine of the C/Rs answered Question D18 of which one C/R answered “Don't know.” Six of the 10 C/Rs followed‐up the recommendation regarding what tooth brush to use and the two C/Rs answering “No” stated that this was done “If the patient had problems” or that this was done by the “Ordinary dentist.” Nine of the C/Rs gave re‐information about toothpaste (Question D19).

D20. Re‐information about recommended interproximal oral self‐care aids (Table 5)

Of the nine C/Rs answering the question, seven said that they gave such information.

All of the nine C/Rs answering Question D21 stated that they give information, instructions, and motivation regarding the importance of mouth opening exercises and that the patient received both oral and written information (Table 5).

D22. Is the patient re‐informed about the importance of abstaining from smoking and alcohol during cancer treatment? (Table 5)

Of the six C/Rs that answered “Yes,” one added “Smoking,” and of the five C/Rs that answered “No,” two C/Rs added “Not from the dental clinic” and “Possibly smoking.”

All C/Rs said that they orally inform the patient about late or chronic oral complications related to cancer treatment and means to relieve such complications and six C/Rs also gave such information in writing (Questions D23a). All C/Rs informed about trismus, osteoradionecrosis, permanent xerostomia, and increased risk of caries (Question D23b). Nine C/Rs informed about oral mucositis, fragile and sensitive mucosal membranes, and increased risk of fungal infections. Other late complications, which the C/Rs informed about were as follows: effects on the sense of smell and taste (eight C/Rs), difficulties in swallowing (seven C/Rs), increased risk of infections in the mucosal membranes (six C/Rs), difficulties in speaking (five C/Rs), and lymphedema (three C/Rs).

### 3.3 After completed cancer treatment

A1a. At which time‐points after completed cancer treatment is the patient followed‐up?

Four C/Rs had a follow‐up at 1 month post treatment, one C/R at 6 weeks post treatment, eight C/Rs at 3 months post treatment, seven C/Rs at 6 months post treatment, one C/R at 9 months post treatment, and three C/Rs at 12 months post treatment. The number of follow‐ups varied between one (three C/Rs) and four (two C/Rs). Examinations and registrations performed at these follow‐ups were the ability to open the mouth (all C/Rs), mucositis, oral hygiene and fluoride use (nine C/Rs), salivary secretion and caries (eight C/Rs), and periodontal status (five C/Rs; Question A1b).

A2. What information is given to the ordinary dental clinic when the patient continue treatment there?

There was variation in the amount of information given and what it consisted of. For example, information was given about the treatment the patient had received and radiation field (two C/Rs), information about an increased risk of caries (three C/Rs), and tooth extractions.

All but one C/R stated that individually tailored oral self‐care instructions were given (Question A3a) and that recommendations regarding products to relieve dry mouth was followed‐up (Question A3b). The 10th C/R commented that such instructions are given by the patient's regular dental clinic.

A4. Is further re‐information, reinstruction, and remotivation regarding oral hygiene given?

Four C/Rs answered “Yes,” two answered “Yes, when needed,” three answered “No,” and two of them commented that “This is done by the patient's regular dental clinic.”

A5. What recommendations are given regarding extra fluoride?

Of the nine C/Rs answering the question, one gave the answer “individually adapted information” and the other eight C/Rs exemplified extra fluoride by suggesting daily rinsing or use of fluoride gel or toothpaste with a high fluoride concentration (5,000 ppm F).

In nine C/Rs, the patient's mouth opening ability at completed treatment is compared with pretreatment values. The 10th C/R commented that this is not done on all patients (Question A6).

A7. For how long after completed radiotherapy is the patient recommended to continue mouth opening exercises?

Many different answers were given, such as the following: “At reduced mouth opening ability” (two C/Rs), “Individual” (one C/R), “At least a year” (one C/R), and “Often lifelong” (one C/R).

A8. What routines do you have regarding invasive treatment in irradiated bone?

All C/Rs stated that a Maxillofacial surgeon is consulted or is remitted to when a tooth needs to be extracted. Seven C/Rs stated that a prophylactic dose of antibiotic is often given when other kinds of invasive treatments need to be done. All C/Rs stated that they have routines for treatment and care at osteoradionecrosis.

### 3.4 Supplementary statements

#### 3.4.1 We have documented procedures for the odontological care

Seven of the nine C/Rs answering that they have documented routines/procedures for the odontological care. Three C/Rs referred to SOM(Swedish Association of Orofacial Medicine (SOM), 2017) and one to Region Skåne's guidelines. (Nilsson & Broberg, 2017) The other three C/Rs referred to regional care programmes/routines not publically available. Two C/Rs did not specify any documents even though they answered “Yes” on the previous question.

## 4 DISCUSSION

In this study, a web‐based questionnaire was filled in by dentists/dental hygienists working in dental clinics responsible for the odontological care of patients undergoing treatment for head and neck cancers in 10 of the 12 C/Rs, which provide treatment for this patient category in Sweden. Four dental hygienists and six dentists completed the questionnaire. The results showed that there are some variations in the data collected about the patients, assessments made regarding the patient's oral status, and large variations regarding advice and recommendations given to the patients regarding their oral health. Plausible explanations are lack of clear guidelines about what means and measures that should be given in connection with cancer treatment. The National Care Programme from 2015 has very limited guidelines(Regional Cancer Centres, Sweden, 2015) regarding odontological care in connection with treatment for cancer of the head and neck. The SOM document(Swedish Association of Orofacial Medicine (SOM), 2017) have some guidelines for odontological treatment and care before and after treatment, but guidelines about during treatment are sparse. Dental personnel therefore have developed their own routines to the best of their knowledge and existing routines in their respective county council/region. Dental personnel in the different county councils/regions probably do not have meetings or conferences with possibilities to discuss the care for the patients.

### 4.1 Methodological considerations

The data collection was performed using an electronic survey to which the respondent was invited to fill in through a web link sent by email. The system “http://Webbenkäter.com” was chosen partly because it is a well‐known system used by many companies throughout the world. Also, this system has a simple and clear layout with the possibility to monitor the questionnaire. The estimated time needed to fill in the questionnaire was 35–40 min, which can be perceived as a long time. Eighteen of the questions had predetermined answering alternatives with the purpose to simplify for the respondent by not having to write the answer in text. However, it might have lead the respondent to mark alternatives which they felt should be performed/checked but which they might not do. The question about information on the importance to avoid smoking and alcohol should have been divided into two questions: one about smoking and one about alcohol. A few questions in the survey were left unanswered by some C/Rs, whereas some C/Rs gave the same answer on several questions, which can be interpreted as time constraints for the respondent or confusion as to what the question concerned.

A positive feature with the web‐based questionnaire was that it was possible to pause it and continue later. This allowed the respondent to bring forth adequate work material (such as routines), which might be needed to answer the questionnaire. A report containing the results was sent by email to all respondents participating in the questionnaire.

### 4.2 Collaboration between dentistry and health care

When we searched for dental clinics involved in the odontological care of patients with head and neck cancers, hospitals in the different county councils/regions were contacted. It was noteworthy that several hospitals had difficulties to give information about which dental clinic/unit was responsible for the odontological care of the patient. Unclarity about who is responsible for the odontological care raises questions on how well the collaboration between dentistry and the healthcare system actually works. Communication between dentistry and healthcare is of great importance and should start immediately when the patient is diagnosed with cancer. (Epstein, Guneri, & Barasch, 2014) Four of the C/Rs stated that they took part in a multidisciplinary conference before the cancer treatment started. All C/Rs stated that several professions were involved in the care of the patient during treatment. It has been shown that such a multi‐professional approach leads to a higher care quality compared with when no multidisciplinary team is involved in the care. (Kelly, Jackson, Hickey, Szallasi, & Bond, 2013)

### 4.3 Routines before treatment

Patient‐related risk factors which may increase the risk of severe mucositis are, for example, poor oral hygiene, periodontal disease, persistent alcohol or tobacco use, xerostomia/hyposalivation, low body mass index (BMI < 18.5), unintentional weight loss, immunosuppression, and being of the female sex. (De Sanctis et al., 2016) It is therefore of great importance to reduce or eliminate as many of those risk factors as possible. (De Sanctis et al., 2016) Patient education in oral hygiene technique is very important as well as a dental examination where preexisting periodontal and dental disease is treated, and including professional dental cleaning. (De Sanctis et al., 2016) The present study showed that the majority of the C/Rs gave patient education in oral hygiene technique. A dental examination prior to starting cancer treatment was performed in all C/Rs and it seemed as if sufficient information about the patients' dental status, as well as their medical status, was obtained. The SOM document, (Swedish Association of Orofacial Medicine (SOM), 2017) which many C/Rs state they base their odontological care on, describes which assessments should be included in the pretreatment dental examination.

It was surprising that only four of the C/Rs measured the stimulated salivary secretion rate. Because saliva is of great importance for oral health, a reduction is well‐known to increase the risk of oral disorders as well as the risk of severe mucositis. (De Sanctis et al., 2016) In our previous study, patients who had a stimulated secretion rate of ≤0.7 ml/min before treatment had a mean secretion rate of 0.1 ml/min at 6 months post treatment, whereas those who had ≥1 ml/min pretreatment had a mean(Almståhl et al., 2018) of 0.6 ml/min. It is therefore likely that patients having a low salivary secretion rate already before starting cancer treatment will have a very low secretion during treatment, which means a high risk of oral complications. Knowledge of the patients' salivary secretion should therefore be taken into consideration when planning the odontological care during cancer treatment.

In the present study, the most common advice given to stimulate the salivary secretion was Xerodent (a lozenge with buffered malic acid; 56%), followed by Proxident spray. If a patient has the capacity to produce saliva, saliva‐stimulating agents should be used. However, if the salivary secretion rate is very low, which is often the case during cancer treatment, it is important to frequently moisturize the oral cavity by using rinses such as saline or saline + bicarbonate. There is no mouth rinse that has been shown to be more effective than others. (De Sanctis et al., 2016) Salt has a mild antimicrobial effect and bicarbonate is the most important buffer component in saliva. It is therefore possible that rinsing with saline and bicarbonate can increase the oral pH, which enhances oral health associated microorganisms and is negative for microorganisms associated with oral mucosal infections. A mouth rinse is effective for rinsing the oral cavity but may not relieve dry mouth problems, wherefore, saliva substitutes should be used. Five of the six C/Rs recommend Proxident spray with sunflower oil and one C/R a saliva substitute mixed at the pharmacy.

Smoking during and after cancer treatment is associated with a weaker treatment result, more side effects, and recurrent cancer. (Fortin, Wang, & Vigneault, 2009; Peppone et al., 2011) According to the American Cancer Society, the intake of alcohol during cancer treatment can irritate mouth sores and make them worse and alcohol can interact with some drugs, which might increase the risk of harmful side effects. In the present study, five C/Rs stated that the patient was informed about the importance of abstaining from smoking and alcohol during cancer treatment. The answers from several C/Rs indicate that dental personal presume that the doctors give this kind of information, especially regarding alcohol. It is important that health care and dental personnel discuss who should give the information so that it is not missed. A higher proportion, 75%, of clinics in Spain recommended the patient to avoid smoking and alcohol during cancer treatment. (Lanzós et al., 2015) Despite recommendation from health care to quit smoking, 35–72% of patients treated for cancer in the head and neck region continues to smoke during and after cancer treatment. (Sharp & Tishelman, 2005) This can be due to the fact that patients seldom are offered professional help to quit. (Sharp & Tishelman, 2005) In the seven different documents, the C/Rs in the present study state that they base their routines on only two write about risks related to smoking and alcohol in connection with cancer treatment. According to the SOM document, (Swedish Association of Orofacial Medicine (SOM), 2017) the use of tobacco and alcohol should be included in the anamnestic questions.

### 4.4 Routines during treatment

De Sanctis et al. (De Sanctis et al., 2016) suggests that an oral examination with evaluation of oral hygiene and assessment of oral mucositis should be performed at least once a week during cancer treatment. Regular visits to the dental hygienist for control and treatment is also suggested in the SOM document. (Swedish Association of Orofacial Medicine (SOM), 2017) In the present study, seven C/Rs stated that the patient visited the dental hygienist at least once a week during cancer treatment. However, three C/Rs did not have this routine, which might increase the risk of both oral complications and patient worry. It is not easy for the patient to know which symptoms or problems are due to the cancer treatment and which are adverse effects of the treatment, and need to be handled by dental professionals. Furthermore, early detection of oral problems may increase the chance of stopping unwanted progression and/or diminish it.

McGuire et al. (McGuire et al., 2013) suggest that oral care protocols including information about the importance of good oral hygiene and frequent rinsing of the oral cavity is important to prevent oral mucositis. Seven C/Rs stated that they recommend good/optimal oral hygiene. The most common rinsing solution recommended was saline (six C/Rs) and Lidocainhydrochloride in oral cleaner (four C/Rs). The use of Lidocainhydrochloride in oral cleaner is suggested in the documents the dentists/dental hygienist state that they base their routines on. However, to the best of the authors' knowledge, there are no studies showing the efficacy of Lidocainhydrochloride in oral cleaner to prevent or relieve oral mucositis.

### 4.5 Routines after completed cancer treatment

Treatment for cancer of the head and neck region may lead to late and/or chronic complications such as persistent xerostomia, low salivary secretion rate, sticky saliva, dysphagia, and trismus. (Almståhl et al., 2019; Deboni et al., 2012; Pauli et al., 2013) Due to decreased bone healing of irradiated bone, there is a risk of osteoradionecrosis, especially in patients who use tobacco or are alcohol abusers. (Epstein et al., 2012) In patients with low salivary secretion rate, there is an increased risk of caries. (Epstein et al., 2012) It is therefore important to follow the patients also after completed cancer treatment. In the present study, there were large variations regarding the number of follow‐up visits and time points for the follow‐ups. The two most common time points for follow‐up was 3 and 6 months post treatment. To the best of the authors' knowledge, there are no guidelines regarding time points for follow‐ups or number of follow‐ups. In the SOM document, (Swedish Association of Orofacial Medicine (SOM), 2017) which several of the respondents said they based their care on, follow‐ups at the ordinary dentist every 3 months during the first year post treatment are suggested. The respondents may have given the time points for follow‐ups at their clinics, and it is likely that the patients are followed‐up also at their ordinary clinics.

As mentioned in the introduction, there are a numerous research articles on guidelines and recommendations for patients undergoing treatment for cancer of the head and neck region. There are several reviews with clinical guidelines and recommendations about means and measures, which should be done before, during, and after treatment to decrease the risk of acute, especially oral mucositis, and late oral complications. (Buglione et al., 2016a; Buglione et al., 2016b; Jensen et al., 2013; Lalla et al., 2014; McGuire et al., 2013; Nicolatou‐Galitis et al., 2013; Sroussi et al., 2017) It is therefore noteworthy that none of the C/Rs mentioned that they base their treatment on research. It should also be noted that three C/Rs stated that they base their treatment on proven experience and some C/Rs have stated recommendations, which are not suggested in the document they base their treatment on. It is important as dental professionals to keep up with the latest research so that current and evidence‐based means and methods are used. It has been shown that evidence‐based prophylactic and therapeutic oral health care can significantly improve the patient's health and quality of life, and reduce health care costs for patients undergoing treatment for cancer. (Epstein et al., 2014)

### 4.6 Ethical considerations

Participation in this questionnaire study was voluntary, and all respondents received written information about the aim of the study. When analysing the data, all C/Rs were given a code from 1 to 10 so that no individual county council or region can be identified. Also, no names of dentists or dental hygienists are given.

### 4.7 Conclusions

The results suggest that there are great variations in the odontological care given to patients undergoing treatment for cancer of the head and neck region in different counties/regions in Sweden. There is a need to develop and implement evidence‐based guidelines to decrease the risk of oral complications and increase both the quality of life and the quality of the care in this patient category.

#### 4.7.1 Scientific rationale for study

Guidelines regarding odontological care for patients undergoing treatment for cancer of the head and neck region are vague and sparse. This may lead to variations in the care given, which in turn may lead to unequal care.

#### 4.7.2 Principle findings

There were large variations in the variables registered and in recommendations to relieve and treat oral mucositis.

#### 4.7.3 Practical implications

There is a need to develop evidence‐based guidelines to improve both the oral and general health for patients with head and neck cancer.

## AUTHOR CONTRIBUTIONS

A. A. conceived the idea. A. A., N. B., and C. K. constructed the questionnaire. N. B. collected the data. All authors analysed the data and co‐wrote the manuscript.

## Supporting information

Data S1.
Supporting Information
