# Supplementary material for: Variations in odontological care routines for patients undergoing treatment for head and neck cancer in county councils/regions of Sweden
Source: Clin Exp Dent Res. 2019 Sep 19;6(1):3–15. doi: 10.1002/cre2.242 (PMC7025979; doi:10.1002/cre2.242)
Supplement: Supplementary file 1 — Data S1. Supporting Information [file CRE2-6-3-s001.pdf]

GÖTEBORGS UNIVERSITET
SAHLGRENSKA AKADEMIN

Odontological care routines for patients undergoing treatment for head and neck cancer

-Survey

Enter the county council/region you work in: *

Profession *

◯ Dentist ◯ Dental hygienist ◯ Dental assistant

◯ Other position:

How many patients/year undergo treatment for cancer in the head and neck region (in your county council /region)?

Part 1. Before starting radiotherapy: Question 1 - 28

(B) 1. How is the contact with the dental care administrated?

Fill in your answer here

(B) 2. Which professions are involved in the care?

- ☐ Dental hygienist
- ☐ Dentist
- ☐ Dental assistant
- ☐ Physician
- ☐ Other care professionals
- ☐ Nurse
- ☐ Speech therapist
- ☐ Dietician
- ☐ No collaboration
- ☐ Contact-nurse
- ☐ Maxillofacial surgeon
- ☐ Counselor

(B) 3. What is recorded in the medical history when the patient visits the dental clinic?

- ☐ Tobacco habits
- ☐ Alcohol habits
- ☐ Allergies
- ☐ Other information
- ☐ Diseases
- ☐ Medicines
- ☐ Oral discomfort
- ☐ Dental fear
- ☐ Regular dentist
- ☐ Contact with dental clinic

(B) 4. Is the patient asked about self-perceived oral health during the examination?

- ☐ Yes
- ☐ No
- ☐ Comments:

(B) 5a. Is information about the risk of acute/temporary oral complications and actions to prevent or relieve them given?

- ☐ In written form
- ☐ Orally
- ☐ Other:

(B) 5b. The patient recieves information about:

- ☐ Oral mucositis
- ☐ Xerostomia
- ☐ Difficulty swallowing
- ☐ Reduced appetite
- ☐ Weight loss
- ☐ Difficulty speaking
- ☐ Pain
- ☐ Mycosis
- ☐ Alterations in taste and smell
- ☐ Fatigue/exhaustion
- ☐ More info:

(B) 6. Is an odontological treatment plan established which the patient and all parties involve agree with before the patient begins cancer therapy?

- [ ] Yes
- [ ] No
- [ ] Comments:

(B) 7 What kinds of examinations are performed before cancer treatment?

- [ ] Extraoral status
- [ ] Intraoral status, examination of the mucosa

X-ray:

- [ ] Complete status
- [ ] Bitewing
- [ ] Orthopantomogram
- [ ] Other:

Periodontal status:

- [ ] Pocket probing depth
- [ ] Furcation involvement
- [ ] Tooth mobility
- [ ] Periradicular status

Caries status:

- [ ] Probing
- [ ] Fluoroscopy
- [ ] Bitewing

Comments:

(B) 8. Is the patient’s prosthetic constructions, it's design and function evaluated?

- [ ] Yes
- [ ] No
- [ ] Comments:

(B) 9. Is previous trauma assessed/considered?

- [ ] Yes
- [ ] No
- [ ] Comments:

(B) 10a. Are photographs taken prior to start of cancer therapy?

- [ ] Yes
- [ ] No
- [ ] Comments:

(B) 10b. Are dental impressions (alginate or similar) made prior to cancer therapy?

- [ ] Yes
- [ ] No
- [ ] Comments:

(B) 11. On which indications are teeth extracted?

Fill in your answer here

(B) 12. How long before the start of the cancer treatment are the extractions performed?

Fill in your answer here

(B) 13. On which indications are caries treatment performed?

Fill in your answer here

(B) 14. Do you have specific routines regarding treatment of carious lesions prior to cancer therapy?

Fill in your answer here

(B) 15. Is the ability to open the mouth determined?

- [ ] Yes
- [ ] No
- [ ] Comments:

(B) 16. Do you have routines regarding reduced ability to open the mouth?

- [ ] Yes
- [ ] No
- [ ] Comments:

(B) 17a. Is the patient's unstimulated salivary secretion rate measured?

- [ ] Yes
- [ ] No
- [ ] Comments:

(B) 17b. Is the stimulated salivary secretion rate measured?

- [ ] Yes
- [ ] No
- [ ] Comments:

(B) 18. Are impressions for customised trays taken, and on what indications?

- [ ] Yes
- [ ] No
- [ ] Comments:

(B) 19a. Does the patient receive an individually adapted recommendation regarding extra fluoride?

- ○ Yes
- ○ No

(B) 19b. If "Yes", state name of preparation, dosage and frequency.

Preparation:

Dosage:

Frequency:

(B) 19c. Does the patient receive an individually adapted recommendation regarding saliva stimulants?

- ○ Yes
- ○ No

(B) 19d. If "Yes", state name of preparation, dosage and frequency.

Preparation:

Dosage:

Frequency:

(B) 19e. Does the patient receive an individually adapted recommendation regarding saliva substitutes?

- ◯ Yes
- ◯ No

(B) 19f. If "Yes", state name of preparation, dosage and frequency.

Preparation:

Dosage:

Frequency:

(B) 20. What dental treatments are given?

- ☐ Scaling
- ☐ Polishing of filling joints
- ☐ Smoothening of sharp cusps and incisor edges
- ☐ Comments:

(B) 21. Does the patient receive information and instruction regarding oral self-care?

- ☐ Yes
- ☐ No
- ☐ Comments:

(B) 22. Is the patient given a motivational dialogue about optimal oral hygiene?

- ☐ Yes
- ☐ No
- ☐ Comments:

(B) 23. Does the patient receive instructions regarding toothbrush technique?

- ☐ Yes
- ☐ No
- ☐ Comments:

(B) 24. Which type of toothbrush do you recommend to the patient?

Fill in your answer here

(B) 25. Which type of toothpaste do you recommend to the patient?

Fill in your answer here

(B) 26. Are individualized recommendations regarding interproximal oral self-care aids given?

- [ ] Yes
- [ ] No
- [ ] State recommendation: ______

(B) 27. Is the patient informed about the importance of abstaining smoking and alcohol during cancer treatment?

- [ ] Yes
- [ ] No
- [ ] Comments: ______

(B) 28. Do you have special routines for patients with reduced ability to maintain adequate oral hygiene or patients with comorbidity?

Fill in your answer here

Part 2. During radiotherapy: Question 1 - 23

(D) 1. How often does the patient meet different professionals during the treatment?

Dental hygienist:

Denstist:

Dental assistant:

Dietician:

Speech therapist:

Nurse:

Physician:

Other personnel:

(D) 2. Is the patient re-informed about the effects of radiation on oral health?

- [ ] Yes
- [ ] No
- [ ] Comments:

(D) 3. How often is the oral cavity inspected by dental professionals?

- [ ] Daily
- [ ] Several times/week
- [ ] Once a week
- [ ] Occasionally per month
- [ ] Other interval:

(D) 4. Which professions outside the dental clinic perform inspection of the oral cavity?

- [ ] Physician
- [ ] Nurse
- [ ] Speech therapist
- [ ] Dietician
- [ ] Other profession:

(D) 5. Are mucositis or other signs of oral disorder noted?

- [ ] Yes
- [ ] No
- [ ] Comments:

(D) 6. What scale is used to grade mucositis?

- [ ] WHO
- [ ] RTOG
- [ ] OMAS
- [ ] CTCAE
- [ ] Other:

(D) 7a. What means are recommended to prevent mucositis?

Fill in your answer here

(D) 7b. What/which preparations are recommended to prevent mucositis?

- [ ] Chlorhexidine
- [ ] Sodium chloride
- [ ] Cortisone
- [ ] Hydrogen peroxide
- [ ] Corsodyl
- [ ] Tap water
- [ ] Hydrocortisone
- [ ] Benzydamine oral rinse
- [ ] Paroex
- [ ] Bromhexine
- [ ] Lidocaine Oral Topical Solution
- [ ] Oral moistener rinse
- [ ] Other preparation:

(D) 8a. What means are recommended to relieve mucositis problems and pain?

Fill in your answer here

(D) 8b. What advice is given regarding pain relief?

Advice:

Preparations:

Other recommendation:

(D) 9. What information, guidelines or research do you base the pain relief treatment regarding oral mucositis on?

Fill in your answer here

(D) 10. Are dietary advice/recommendations given?

- [ ] Yes
- [ ] No

Description of advice:

- [ ]

(D) 11. Which professions give advice about diet?

- ☐ Dental hygienist
- ☐ Physician
- ☐ Dental assistant
- ☐ Dietician
- ☐ Dentist
- ☐ Nurse
- ☐ Speech therapist
- ☐ Other profession:

(D) 12. Are samples for microbial analysis taken at a suspected mucosal infection?

- ☐ Yes
- ☐ No
- ☐ Comments:

(D) 13. Is the fluoride prophylaxis followed up?

- ☐ Yes
- ☐ No
- ☐ Comments:

(D) 14. Is the patient's salivary secretion rate measured during treatment?

- ☐ Yes
- ☐ No
- ☐ Comments:

(D) 15. Which products are recommended at dry mouth?

- ☐ Xerodent
- ☐ Sugar-free chewing gum
- ☐ Salivin lozenge
- ☐ DentiProlozenge Ekulf
- ☐ Fuktisar lozenge
- ☐ Flux Drops lozenge
- ☐ Flux Dry Mouth Gel
- ☐ Zendium Saliva Gel
- ☐ DentiPro gel
- ☐ Proxident gel
- ☐ Proxident mouth-spray
- ☐ Proxident toothpaste
- ☐ Zendium toothpaste
- ☐ Biotène toothpaste
- ☐ GUM Hydral toothpaste
- ☐ Dentosal toothpaste
- ☐ Salutem toothpaste
- ☐ Sensodyne toothpaste
- ☐ TopDent fluoride-gel
- ☐ Duraphat-toothpaste
- ☐ Other recommendation: ____________________

(D) 16. Does the patient receive written information about the recommended preparations?

- ☐ Yes
- ☐ No
- ☐ Comments: ____________________

(D) 17. Is a re-instruction regarding tooth-brushing technique given?

- ☐ Yes
- ☐ No
- ☐ Comments: ____________________

(D) 18. Is the patient given re-information about recommended tooth-brush?

- ☐ Yes
- ☐ No
- ☐ Comments: ____________________

(D) 19a. Is the patient given re-information about tooth-paste?

- ◯ Yes
- ◯ No

(D) 19b. If "Yes", specify taste, dosage and frequency.

Taste:

Dosage:

Frequency:

(D) 20. Re-information about recommended interproximal oral self-care aids?

- ☐ Yes
- ☐ No
- ☐ Comments:

(D) 21. Is information, instruction and motivation given about mouth-opening exercises?

- ☐ Orally
- ☐ Written info
- ☐ Other:

(D) 22a. Is the patient given re-information about the importance of abstaining smoking and alcohol during cancer treatment?

- ☐ Yes
- ☐ No
- ☐ Comments:

(D) 22b. If "Yes":

How often is the patient's smoking habits followed up?

How often is alcohol habits followed up?

(D) 23a. Do you give information about the risk of late/chronic complications and means to relieve them?

- ☐ Written info
- ☐ Orally
- ☐ Other

(D) 23b. The patient is informed about:

☐ Oral mucositis	☐ Lymphatic edema	☐ Difficulty speaking	☐ Increased risk of oral mucosal infection
☐ Trismus	☐ Permanent Xerostomia	☐ Altered taste and smell	☐ Increased risk for caries
☐ Osteoradionecrosis	☐ Difficulty swallowing	☐ Fragile/sensitive mucous membrane	☐ Increased risk of mycosis

☐ Other information:

Part 3. After radiotherapy: Question 1 - 9

(A) 1a. At what time-points after completed cancer treatment is the patient followed up?

☐ 1 month ☐ 3 month ☐ 6 month ☐ 9 month ☐ 12 month ☐ 24 month

☐ Other timeframe:

(A) 1b. Which examinations and registrations are made during these follow-ups?

- [ ] Mouth opening ability
- [ ] Oral mucositis
- [ ] Salivary secretion rate
- [ ] Oral hygiene
- [ ] Caries
- [ ] Use of fluoride
- [ ] Periodontitis
- [ ] Other:

(A) 2. What information is given to the ordinary dental clinic when the patient continue treatment there?

Fill in your answer here

(A) 3a. Are individually tailored oral hygiene self care instructions given to the patient?

- [ ] Yes
- [ ] No
- [ ] Comments:

(A) 3b. Are the recommended preparations for relieving dry mouth followed up?

- [ ] Yes
- [ ] No
- [ ] Comments:

(A) 4. Is further re-information, re-instruction and re-motivation regarding oral hygiene given?

- [ ] Yes
- [ ] No
- [ ] Comments:

(A) 5. What recommendations are given regarding extra fluoride?

Fill in your answer here

(A) 6. Is the difference in the patient's maximum mouth opening ability after completed radiation therapy, compared with the mouth opening ability pre-radiation treatment?

- [ ] Yes
- [ ] No
- [ ] Comments:

(A) 7. For how long after completed radiation therapy is the patient recommended to continue mouth opening exercises?

Fill in your answer here

(A) 8. What routines do you have regarding invasive treatment in irradiated bone?

Fill in your answer here

(A) 9. Do you have routines for treatment and care of osteoradionecrosis?

Fill in your answer here

Supplementary statements/question:

We have documented procedures for the odontological care

◯ Yes ◯ No ◯ Do not know

If "Yes" to the above question, describe what forms the basis for these routines (eg Guidelines, scientific studies)
